# Résultats et limites dans la chirurgie des lipœdèmes, lymphœdèmes et éléphantiasis des membres inférieurs dans un pays à ressources limitées: République démocratique du Congo, 2010 à 2025

**DOI:** 10.11604/pamj.2025.52.88.49056

**Published:** 2025-10-30

**Authors:** Anatole Kibadi-Kapay

**Affiliations:** 1 Service de Chirurgie Plastique Reconstructive, Esthétique et Chirurgie de la Main, Cliniques Universitaires de Kinshasa, Faculté de Médecine, Université de Kinshasa, Kinshasa, République Démocratique du Congo

**Keywords:** Lymphœdèmes, lymphœdèmes, éléphantiasis, membres inférieurs, chirurgie, pays à ressources limitées, Lipoedema, lymphedema, elephantiasis, lower limbs, surgery, resource-limited countries

## Abstract

Cette étude vise à présenter les résultats et les limites de la chirurgie des lipœdèmes et des lymphœdèmes des membres inférieurs dans un environnement chirurgical à ressources limitées. Il s’agissait d’une étude transversale, analytique, à récolte prospective et intra-hospitalière, couvrant une période de 15 ans. Nous avons traité chirurgicalement 119 patients dont 18 patients avec lipœdème (15,12%), 69 avec lymphœdème stade 2 (57,1%) et 32 avec éléphantiasis (26,8%). Nous avons eu 81 femmes (68%) et 38 hommes (31,9%). La majorité (57,7%) des patients avec lymphœdème était dans la tranche d'âge de 30 à 49 ans. La chirurgie plastique et d´exérèse a été réalisée chez 42,2% des patients avec lymphœdèmes de stade 2 et chez 87,5% de stade 3 (éléphantiasis). Quant à la chirurgie des lipœdèmes, elle a consisté essentiellement en une liposuccion (77,7%). Aucun acte de reconstruction à l’échelle du réseau lymphatique n’a été réalisé. Nos résultats, à 2 ans de recul post-chirurgie, étaient très satisfaisants avec une symétrie du membre chez 83,3% des patientes opérées pour lipœdème, chez 84,2% des patients opérés pour lymphœdème stade 2 et chez 85% des patients opérés pour éléphantiasis. La chirurgie des lipœdèmes, lymphœdèmes et éléphantiasis des membres inférieurs dans un pays à ressources limitées est possible, bien que des défis subsistent. L’absence de la reconstruction à l’échelle du réseau lymphatique constitue nos limites. La pratique de la microchirurgie s’avère nécessaire pour un résultat optimal.

## Abstract (en)

This study aims to present the results and limitations of surgery for lower limb lipoedema and lymphedema in a resource-limited surgical setting. This was a cross-sectional, analytical, prospective, in-hospital study covering 15 years. We surgically treated 119 patients, including 18 with lipoedema (15.1%), 69 with stage 2 lymphedema (57.9%), and 32 with elephantiasis (26.9%). We included 81 women (68%) and 38 men (31.9%). The majority (57.7%) of patients with lymphedema were between 30 and 49 years of age. Plastic and excisional surgery was performed in 42.2% of patients with stage 2 lymphedema and in 87.5% with stage 3 (elephantiasis). Lipoedema surgery consisted primarily of liposuction (77.7%). No lymphatic network reconstruction was performed. Our results, at 2 years’ post-surgery, were very satisfactory, with limb symmetry in 83.3% of patients operated on for lipoedema, in 84.2% of patients operated on for stage 2 lymphedema, and in 85% of patients operated on for elephantiasis. Surgery for lower limb lipoedema, lymphedema, and elephantiasis is feasible in a resource-limited setting, although challenges remain. The lack of lymphatic network reconstruction constitutes our limitations. Microsurgery is necessary for optimal results.

## Introduction

Le terme lymphœdème se décompose en deux mots: la lymphe qui vient du latin *lympha* (eau, fontaine) et œdème qui vient du grec *οἴδημα, oídêma* (gonflement, tumeur). Le lymphœdème est une accumulation de lymphe dans l’espace interstitiel causée par un dysfonctionnement du système lymphatique, et qui engendre une stase du liquide lymphatique, une augmentation du volume du membre concerné, une inflammation et une accumulation de tissu adipeux [1-4]. Il existe trois stades évolutifs de lymphœdème avec une gravité croissante: le stade 1 «hydrique» qui correspond à un lymphœdème partiel qui tend à s'estomper la nuit; le stade 2 «fibreux» qui correspond à un lymphœdème persistant, positif au test basé sur le signe de Stemmer et avec un début d´altération de la peau; le stade 3 qui correspond au stade de l'éléphantiasis [5].

Le lipœdème des membres inférieurs, quant à lui, est une maladie chronique caractérisée par une accumulation anormale de graisse sous la peau, touchant principalement les jambes et les cuisses, tout en épargnant les pieds. Cette accumulation entraîne une augmentation disproportionnée du volume de ces membres, souvent de manière symétrique et bilatérale [6,7]. Le lipœdème peut s'accompagner de douleurs et de sensibilité accrue à la pression [6,7].

La chirurgie de lymphœdème stade 2 et stade 3 (éléphantiasis) comporte trois procédés chirurgicaux suivants: la chirurgie de reconstruction faisant appel à la microchirurgie à l'échelle du réseau lymphatique [8,9]; la chirurgie plastique et d'exérèse correspondant à une réduction de l'excès de tissu cutané et sous-cutané après réussite de la thérapie décongestive complexe [10-12]; et la liposuccion consistant à réduire le stock de graisses, de la lymphe par aspiration [13,14]. Quant à la chirurgie du lipœdème, elle consiste essentiellement en lipoaspiration [15,16].

Les lymphœdèmes et les lipœdèmes des membres inférieurs affectent des millions de personnes dans le monde et ils constituent à l’heure actuelle un réel problème de santé dans les pays à ressources limitées [11]; et à cause des infections liées aux maladies tropicales, de la mobilité limitée des patients, du retentissement psychosocial chez les patients. L'objectif de ce travail est de présenter nos résultats et nos limites dans un environnement chirurgical à ressources limitées.

## Méthodes

**Type d’étude, cadre, période et patients: il s’agissait d’une** étude transversale, analytique, à récolte prospective et intra-hospitalière, menée du 1^er^ juin 2010 au 1^er^ juin 2025. Notre population d´étude était constituée de patients que nous avons suivis et traités dans différentes institutions hospitalières du secteur public et privé à Kinshasa en République Démocratique du Congo (RDC), Cliniques Universitaires de Kinshasa, Centre Médical de Kinshasa, Centre Médical Diamant, HJ Hospital, Desmet Hôpital de Kinsuka, Hôpital du Cinquantenaire, Centre Médical de la direction générale des impôts (DGI), répondant à nos variables d'étude.

Variables d’étude : les données socio-démographiques concernaient: l’âge, le sexe et le délai de consultation évalué à partir de la durée écoulée entre l’apparition du premier symptôme constaté par le patient et notre prise en charge. Les données diagnostiques étaient: les antécédents des patients (famille, puberté, grossesse, insuffisance veineuse); l'atteinte unilatérale ou bilatérale des membres; le diagnostic clinique (lipœdème, lymphœdème stade 2, lymphœdème stade 3 ou éléphantiasis); l'étiologie du lymphœdème (primaire, secondaire); le type de chirurgie réalisée; le résultat à deux de recul post-chirurgie.

**Préparation du malade avant la chirurgie:** elle était de toute première importance. et comportait: 1) une désinfection soigneuse et prolongée de la peau, des replis, des sillons qui étaient des refuges des germes. Le savonnage et la désinfection aux antiseptiques étaient répétés plusieurs jours avant l'intervention; 2) le repos au lit prolongé, membre soulevé pour faire diminuer au maximum l'œdème. Ceci demandait au moins 4 semaines; 3) la lutte contre l'infection, en présence des poussées de lymphangite à répétition, la désinfection cutanée avec l'association aqueuse chloramine-métronidazole-furandoine «Solution Kibadi» [17]. Si indiqué, les antibiotiques administrés avant l'intervention étaient choisis après antibiogramme guidé par des prélèvements.

**Type de chirurgie réalisée:** les patients avec lipœdème, de lymphœdème stade 2, d'éléphantiasis ont été traités chirurgicalement par l'un des cinq procédés chirurgicaux suivants: 1) liposuccion; 2) dermolipectomie; 3) chirurgie plastique et d'exérèse; 4) liposuccion et dermolipectomie; 5) liposuccion associée à la chirurgie plastique et d'exérèse.

## Résultats

Pour une période de 15 ans, soit du 1^er^ juin 2010 au 1^er^ juin 2025, nous avons traité chirurgicalement 119 patients pour lipœdème, lymphœdème stade 2 et éléphantiasis. Nous avons eu une moyenne annuelle de 7,9 malades opérés. Nous avons noté 81 femmes (68%) et 38 hommes (31,9%). Nous avons traité 18 patients avec lipœdème (15,12%); 69 avec lymphœdème stade 2 (57,1%) et 32 avec éléphantiasis (26,8%).

L'atteinte des membres inférieurs était unilatérale ou bilatérale. Pour les lipœdèmes, l'atteinte était unilatérale chez 2 patients sur 18 (11,1%) et bilatérale chez 16 patients sur 18 (88,8%). L'atteinte était unilatérale chez 48 patients sur 69 (69,5%) pour le lymphœdème au stade 2 et chez 27 patients sur 32 (84,3%) pour le lymphœdème au stade 3 (éléphantiasis). Elle était bilatérale chez 21 patients sur 69 (30,4%) pour le lymphœdème stade 2 et chez 5 patients sur 32 (15,6%) pour le lymphœdème stade 3 (éléphantiasis).

Les lymphœdèmes stade 2 et stade 3 (éléphantiasis) concernent 101 patients, soit 84,8% de notre population d'étude. Le Tableau 1 présente les effectifs de patients avec des lymphœdèmes des membres inférieurs par tranche d'âge ainsi que leur étiologie. La tranche d'âge de 30 à 49 ans a représenté 57,7% des patients avec des lymphœdèmes. La moyenne d'âge était de 40,2 ans. Dans les antécédents, nous avons retrouvé la notion de surpoids dans 77,1% et de l'insuffisance veineuse chronique dans 23,6% des cas. La durée d'évolution de plus de 12 mois a représenté 52,5%. La durée d'évolution de 6 à 12 mois a représenté 27,4% des cas et la durée d´évolution de moins de 6 mois a représenté 20,1% des cas.

**Tableau 1:** effectifs de patients avec lymphœdèmes par tranche d'âge et leur étiologie

Effectifs de patients par tranches d'âge	Etiologie			
	Primaire	Secondaire		
		Infections (Erysipèle, DHB-FN, lymphangite, filariose)	Traumatisme	Exérèse chirurgicale
≤ 29 ans: 14 sur 101 patients (13,8 %)	11 sur 14 patients (78,5 %)	3 sur 14 patients (21,4 %)	-	-
30 à 49 ans: 58 patients sur 101 (57,7 %)	13 sur 58 patients (22,4 %)	38 sur 58 patients (65,5 %)	5 sur 58 patients (8,6 %)	2 sur 58 patients (3;4 %)
50 à 69 ans: 24 patients sur 101 (23,7 %)	4 sur 24 patients (18,5 %)	12 sur 24 patients (50 %)	5 sur 24 patients (20,8 %)	3 sur 24 patients (12,5 %)
≥ 70 ans: 5 patients sur 101 (4,7 %)	-	3 sur 5 patients (60 %)	1 sur 5 patients (20 %)	1 sur 5 patients (20 %)
Total: 101 patients	28 sur 101 patients (27,5 %)	56 sur 101 patients (52,7 %)	11 sur 101 patients (10,8 %)	6 sur 101 patients (5,9 %)

Quant aux 18 patientes avec lipœdèmes opérées, elles étaient toutes de sexe féminin et obèses (100%). Seize patientes (88,8%) avaient des antécédents de lipœdèmes de jambes dans la famille; 15 patientes (83,3%) ont développé le lipœdème pendant ou après la puberté; 12 patientes (66,6%) présentaient une insuffisance veineuse et 2 patientes (11,1%) ont développé les symptômes autour de la grossesse. L'atteinte des membres inférieurs était unilatérale dans 11,1% et bilatérale dans 88,8% des cas.

**Figure 1:**
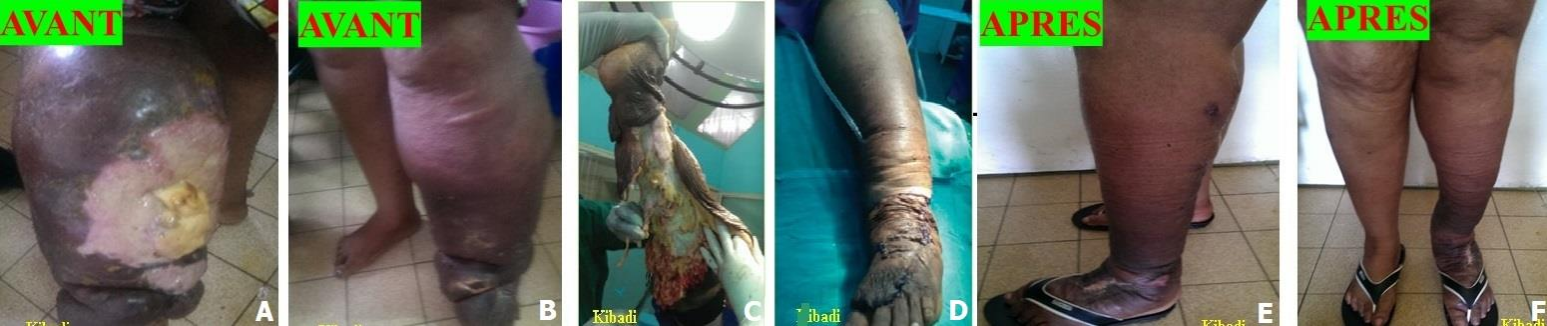
A, B, C, D, E, F) lymphœdème stade 3 ulcéré de la jambe gauche traitée par fascio-lymphangiectomie avec conservation de la peau

**Figure 2:**
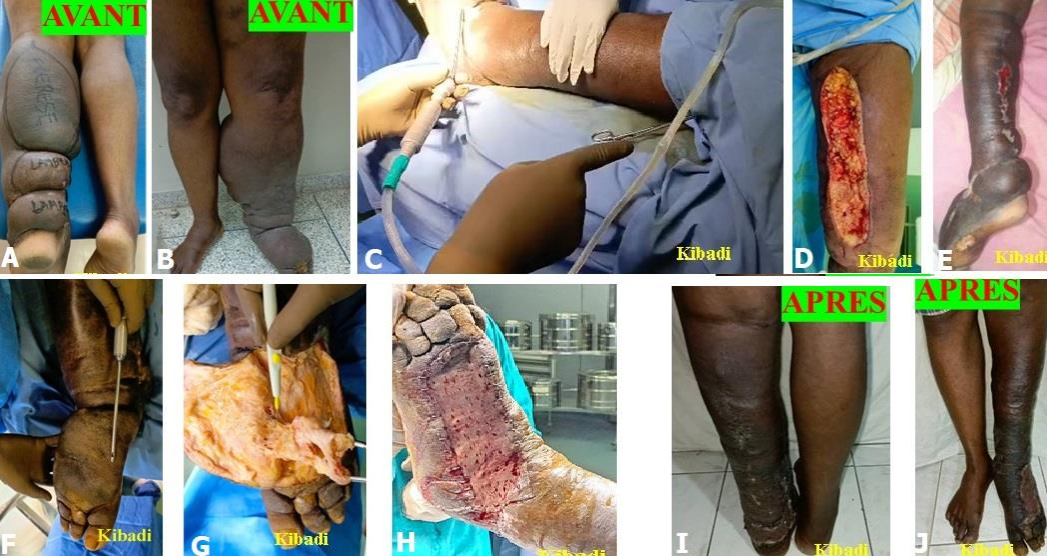
A, B, C, D, E, F, G, H, I, J) lymphœdème stade 2 de la jambe et stade 3 du pied gauche traité par lympholipoaspiration, fascio lymphangiectomie et greffe cutané

**Figure 3:**
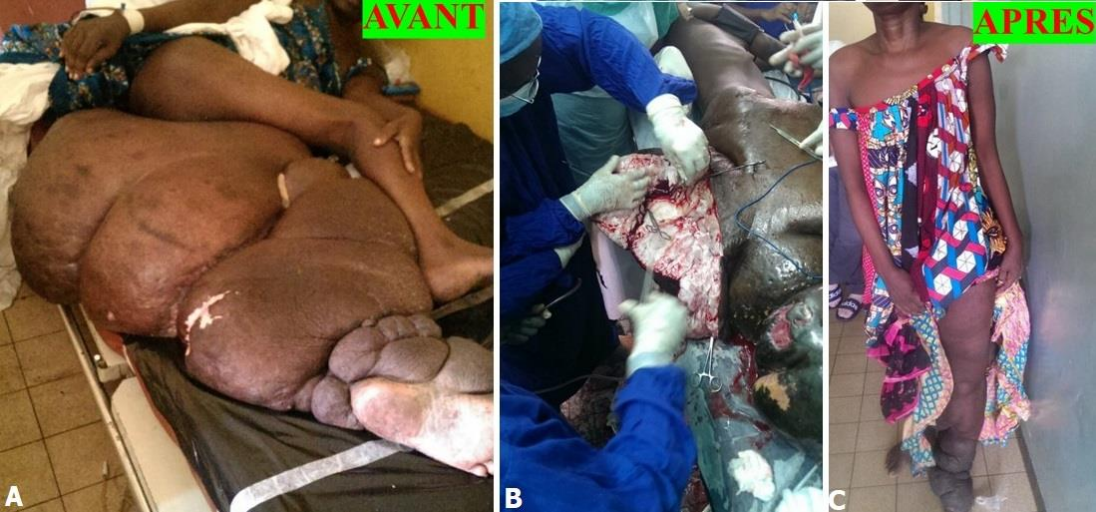
A, B, C) monstrueux éléphantiasis du membre inférieur gauche traité par des procédés de chirurgie plastique et d'exérèse

**Figure 4:**
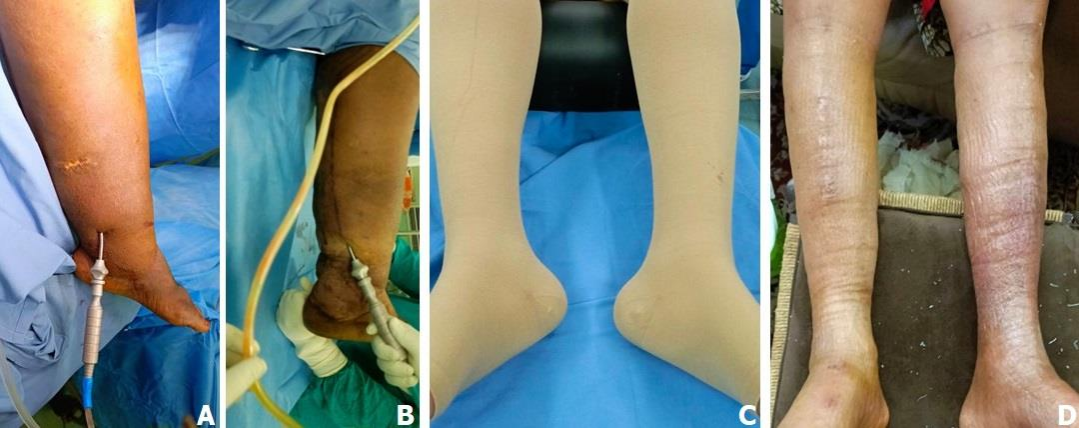
A, B, C, D) lipœdèmes traités par lipoaspiration et port des vêtements compressifs après chirurgie

Nous avons traité chirurgicalement 119 patients avec lipœdème, lymphœdème stade 2 et éléphantiasis par l’un des cinq procédés chirurgicaux suivants: 1) liposuccion; 2) dermolipectomie; 3) chirurgie plastique et d’exérèse ; 4) liposuccion et dermolipectomie; 5) liposuccion associée à la chirurgie plastique et d’exérèse. Sur les 69 patients avec lymphœdème stade 2, 39 patients (56,5%) ont bénéficié d'une chirurgie plastique et d'exérèse, 20 patients (28,9%) d'une liposuccion associée à la chirurgie plastique et d'exérèse, et 10 patients (14,4%) d’une liposuccion. La Figure 1 illustre une patiente ayant bénéficié d'une chirurgie plastique et d'exérèse et la Figure 2, un patient traité par la liposuccion associée à la chirurgie plastique et d'exérèse. Sur les 32 patients avec éléphantiasis, 28 patients (87,5%) ont bénéficié d'une chirurgie plastique et d'exérèse, et 4 patients (12,5%) d'une liposuccion associée à la chirurgie plastique et d'exérèse. La Figure 3 illustre une patiente présentant un monstrueux éléphantiasis du membre inférieur gauche traitée par la chirurgie plastique et d'exérèse. Sur les 18 patientes opérées pour lipœdèmes, 14 patientes (77,7%) ont bénéficié d'une liposuccion, 3 patientes (16,6%) d'une liposuccion avec dermolipectomie, et 1 patiente (5,5%) d'une dermolipectomie uniquement. La Figure 4 présente des patientes avec lipœdèmes des membres inférieurs traitées par la liposuccion.

Nos résultats, à au moins 2 ans de recul post-chirurgie, étaient très satisfaisants avec une symétrie du membre dans la majorité des cas. Elle était observée chez les 10 patientes de 12 revues (83,3%) sur les 18 opérées avec lipœdèmes, chez les 32 patients de 38 revus (84,2%) sur les 69 opérés avec lymphœdème stade 2, et chez les 17 patients de 20 revus (85%) sur les 32 opérés avec éléphantiasis. À deux ans de recul post-thérapeutique, nous avons revu seulement 70 patients sur les 119 opérés (58,8%) et nous avons perdu de vue 49 patients sur les 119 opérés (41,1%).

## Discussion

**Caractéristiques générales de la population étudiée:** nous avons traité chirurgicalement 119 patients dont 68% des femmes et 31,9% des hommes. Nos résultats sont inférieurs à ceux d'Ouled au Maroc qui a trouvé une nette prédominance féminine de 75,8% [18]. Nos résultats sont supérieurs à ceux de Gassama *et al*. au Mali avec une prédominance féminine de 65,5% des cas [3]. Nos résultats sont donc proches des données de la littérature selon lesquelles les lymphœdèmes atteignent les femmes dans 2/3 des cas [19]. En effet, les données publiées montrent que le lymphœdème touche plus les femmes que les hommes. Allen *et al*. [20], sur une série de 300 cas, ont montré une nette prépondérance féminine allant jusqu'à 87%. Kinmonth *et al*. ont publié une étude composée de 107 cas et ils ont confirmé la prédominance féminine avec 72% [21]. Allen rapporté par Brunner, sur une série de 536 cas, a montré une large différence des femmes (87%) aux hommes (13%) [22].

Dans notre série pour les lymphœdèmes, la tranche d'âge de 30 à 49 ans était majoritaire et elle a représenté 57,7% des patients avec une moyenne d'âge de 40,2 ans. Dans la série de Gassama *et al*. la tranche d'âge de 31 à 50 ans a représenté 53,61% avec une moyenne d'âge de 36,74 ans. Nos résultats sont différents de ceux de Bongard [2] et de la littérature qui affirment que les lymphœdèmes surviennent avant l'âge de 35 ans. Dans notre série, cela pourrait s'expliquer par la prédominance du lymphœdème secondaire qui représentait 72,5% des cas. Dans notre série, sur 101 patients avec lymphœdème de stade 2 et éléphantiasis, 56 patients (52,7%) présentaient des lipœdèmes secondaires à une infection (érysipèle, lymphangite). Un élément déclencheur est souvent à l'origine de l'apparition d'un lymphœdème secondaire [21]. L'érysipèle est majoritairement retrouvé au niveau des membres inférieurs. La porte d'entrée se fait souvent par le biais d'une lésion dermatologique ou bien d'une plaie [22,23]. Dans notre série, nous avons observé 27,5% de lymphœdèmes primaires. Le lymphœdème primaire est causé par des anomalies d'origine génétique, plus de 20 gènes différents pouvant être responsables d'anomalies liées au système lymphatique (VEGFR-3, CCBE1, FOXC2, SOX18, GATA2, GJC2, GJA1, PTPN11, SOS1, KIF11, AKT1) (18). D'un point de vue étiologique, il y a un dysfonctionnement du drainage lymphatique qui est dû à une malformation vasculaire, causée par une mutation génétique (maladie de Milroy) qui provoque la formation d’œdèmes riches en cellules mononucléées [23].

### Nos résultats de la chirurgie des lymphœdèmes stade 2 et stade 3 (éléphantiasis)

**Traitement actuel des lymphœdèmes:** à l'heure actuelle, il n'existe pas de traitement curatif du lymphœdème. Le traitement actuel repose principalement sur la thérapie décongestive lymphatique (TDL), qui comprend le drainage lymphatique manuel, la compression, les soins de la peau et des exercices spécifiques. La chirurgie reste une option intéressante dans la prise en charge du lymphœdème stades 2 et 3 car elle permet d’améliorer significativement l’état du lymphœdème et d’apporter un confort considérable au patient. La chirurgie se classe en trois catégories: la chirurgie préventive, la chirurgie curative et la chirurgie palliative. Cependant, trois procédés chirurgicaux existent: 1) la chirurgie de reconstruction faisant appel à la microchirurgie à l'échelle du réseau lymphatique; 2) la chirurgie plastique et d'exérèse correspondant à une réduction de l'excès de tissu après réussite de la thérapie décongestive complexe; 3) la liposuccion consistant à réduire le stock de graisse, de la lymphe par aspiration [24-28].

**Chirurgie des lymphœdèmes stade 2 et de stade 3 (éléphantiasis):** le traitement chirurgical des lymphœdèmes et de l'éléphantiasis peut avoir un double but: 1) faciliter le drainage de la lymphe en cherchant à créer des voies lymphatiques artificielles; ce sont les opérations de drainage; 2) réséquer toute la masse des tissus malades, en particulier du tissu sous-cutané hypertrophique et, dans certains cas, y compris la peau si elle est de mauvaise qualité. Ce sont les opérations plastiques et d'exérèse.

Nonobstant les modifications de détails apportées par chaque auteur, nous pouvons classer les opérations de chirurgie plastique et d'exérèse, par ordre d´importance de la résection, en deux grands groupes: les résections partielles (limitées en largeur) et les résections totales enlevant le tissu sous-cutané et l'aponévrose (fascia) sur toute la largeur du membre malade œdématié. Les résections totales comportent deux types, suivant que la peau est conservée ou non: 1) l'aponevro-lymphangiectomie superficielle ou fascio-lymphangiectomie superficielle sous cutanée qui conserve la peau; 2) l'aponeuro-lymphangiectomie cutanée totale ou fascio-lymphangiectomie cutanée totale avec excision du tissu sous-cutané, de la peau et de l'aponévrose (fascia), avec mise en place des greffes prélevées soit sur le membre lui-même, soit à distance.

**Nos résultats de chirurgie plastique et d’exérèse dans les lymphœdèmes stade 2 et dans les éléphantiasis:** dans notre série pour les lymphœdèmes, la chirurgie plastique et d'exérèse a concerné 42,2% de lymphœdèmes stade 2 et 87,5% de lymphœdèmes stade 3 (éléphantiasis). Cette chirurgie a consisté en une réduction de l´excès de tissu après réussite de la thérapie décongestive complexe. La chirurgie a consisté en une lymphangiectomie avec conservation cutanée. Cette chirurgie, appelée aussi la dermolipectomie avait pour objectif de rendre au membre touché une forme convenable qui se rapprochait de la normalité [29,30]. Dans notre série, nous avons également réalisé une dermolipectomie à la suite d'une liposuccion chez 15% des patients avec lymphœdèmes stade 2 et chez 14,4% des patients avec éléphantiasis; lorsqu'il résidait un excès tissulaire après le retrait d´une importante masse graisseuse, pour donner à la peau un aspect plus esthétique.

**Nos résultats de liposuccion dans les lipœdèmes:** dans notre série, la chirurgie des lipœdèmes a consisté essentiellement en une liposuccion (77,7%). Les techniques de liposuccion [30] consistaient premièrement à placer un garrot à la racine du membre concerné et nous réalisions une infiltration d'un sérum adrénaliné. Dans un second temps, l'aspiration a été réalisée de la zone distale vers la zone proximale du membre en question via une canule de 3 mm. Dans les suites opératoires, il était primordial de porter un vêtement de compression afin d’éviter l'effet rebond du lipœdème. Cette chirurgie était nécessaire car ni le drainage ni la compression ne pouvaient réduire ce dépôt de graisse. Dans ce cas, c'était la masse graisseuse qui était majoritaire par rapport à la masse liquidienne qui, elle, répondait positivement aux traitements par compression.

Nos résultats, à au moins 2 ans de recul post-chirurgie, étaient très satisfaisants. Nous avons obtenu une symétrie du membre dans la majorité des cas, chez 83,3% des patientes opérées pour lipœdeme, chez 84,2% des patients opérés pour lymphœdème stade 2 et chez 85% des patients opérés pour éléphantiasis. Néanmoins, une compression médicale était nécessaire sur le long terme pour avoir un résultat concluant. Mais, nous n'avons évalué que 49 patients sur les 119 opérés (41,1%) et (58,9%) de patients étaient perdus de vue, après deux ans post-chirurgie. Plusieurs facteurs peuvent expliquer la perte de vue de nos patients après chirurgie. Ces raisons incluent des difficultés d'accès aux soins, des problèmes de suivi post-opératoire, des complications liées à l'opération, des facteurs socio-économiques et de la mobilité des patients.

**Limites:** mis à part les limites liées aux raisons non chirurgicales (accès limité aux soins, facteurs socio-économiques, la pauvreté de la population), nos limites peuvent se résumer à l’absence de la pratique de microchirurgie dans les traitements de lymphœdèmes. En effet, dans notre série, sur les 119 patients opérés pour lymphœdèmes, aucun d'eux n'a bénéficié d'une reconstruction à l'échelle du réseau lymphatique. Et pourtant, le traitement microchirurgical des lymphœdèmes est fréquent à nos jours [31,32]. La procédure de la reconstruction lymphatique commence en amont de l´intervention pour une observation du système lymphatique (lymphofluoroscopie) par injection du vert d´indocyanine capté par une caméra infrarouge dans les vaisseaux et ainsi obtenir une cartographie du réseau [31,32]. Dans un second temps, après avoir obtenu une vision précise du système lymphatique, quatre techniques sont envisageables par le chirurgien [31,32]. L’anastomose: il s'agit de connecter un vaisseau lymphatique à une veine (anastomose lymphoveineuse) ou un ganglion lymphatique à une veine (anastomose lymphatico-veinulaire). La veine d’interposition : il s’agit d’utiliser une veine saine pour créer un pont entre deux collecteurs lymphatiques. Cette technique permet de traiter une obstruction. Le transfert autologue ganglionnaire: il s’agit de prélever des ganglions sains et de les positionner dans des zones endommagées ou non fonctionnelles. Le transfert autologue des vaisseaux lymphatiques: il s'agit de prélever des vaisseaux lymphatiques sains et de les positionner dans des zones endommagées ou non fonctionnelles.

De nos jours, l'utilisation de la robotique en chirurgie de l'anastomose lympho-veineuse (ALV) a aussi montré des résultats prometteurs lors d'études cliniques [33]. En effet, deux systèmes chirurgicaux utilisés en pratique robotique clinique ont été identifiés: le microsure (MUSA) et le système chirurgical Symani (micro-instruments médicaux) [33]. L'assistance robotique peut contribuer à améliorer les capacités techniques du chirurgien pour les traitements des lymphœdèmes des membres inférieurs grâce à la mise à l'échelle des mouvements et à la filtration des tremblements, facilitant ainsi les étapes les plus délicates de l'ALV. La courbe d´apprentissage est abrupte, et cette technique pourrait permettre de rendre les reconstructions microchirurgicales accessibles à un plus grand nombre de patients [33]. Des développements ultérieurs pourraient inclure un retour haptique, des programmes de formation structurés et une optimisation des coûts grâce à la diffusion de la technologie, très utiles pour nos pays à ressources limitées.

**Éthique:** la recherche a été menée conformément à la réglementation en vigueur concernant les principes éthiques de la déclaration d'Helsinki.

**Consentement éclairé et informations sur les patients:** l'auteur déclare que ce rapport ne contient aucune information personnelle permettant d'identifier les patients.

## Conclusion

Nos résultats à deux ans de recul post-chirurgie sont très satisfaisants car les patients retrouvent dans la majorité des cas une symétrie du membre. La présente étude révèle que la chirurgie des lipœdèmes, lymphœdèmes et éléphantiasis des membres inférieurs dans un pays à ressources limitées est possible, bien que des défis subsistent. L'absence de la reconstruction à l’échelle du réseau lymphatique constitue nos limites. La microchirurgie s'avère nécessaire pour un résultat optimal.

### Etat des connaissances sur le sujet

- Liposuccion pour lipœdèmes, chirurgie de réduction pour lymphœdèmes stade 2 et stade 3;
- Microchirurgie pour différents stades de lymphœdèmes.

### Contribution de notre étude à la connaissance

- Données statistiques disponibles, chirurgie possible des lipœdèmes, lymphœdèmes et éléphantiasis dans un pays à ressources limitées;
- Défi dans la reconstruction à l’échelle du réseau lymphatique.

## Remerciements

Nos sincères remerciements s'adressent aux différents staffs médicaux, anesthésiques et infirmiers, qui ont contribué à la prise en charge des patients, des Cliniques Universitaires de Kinshasa, du Centre Médical de Kinshasa, du Centre Médical Diamant, de HJ Hospital, de Desmet Hôpital de Kinsuka, de l'Hôpital du Cinquantenaire, du Centre Médical de la DGI.
